# Evaluation of a novel fetal echocardiography training programme in two tertiary care obstetric Centres

**DOI:** 10.1002/ijgo.71091

**Published:** 2026-05-19

**Authors:** Fiona Cody, Orla Franklin, Caoimhe Lynch, Zara Molphy, Patrick Dicker, Fergal Malone, Fionnuala M. Breathnach

**Affiliations:** ^1^ Department of Obstetrics and Gynecology Royal College of Surgeons in Ireland (RCSI) Dublin Ireland; ^2^ Department of Obstetrics and Gynecology Rotunda Hospital Dublin Ireland; ^3^ Department of Pediatric Cardiology Children's Health Ireland CHI@Crumlin Dublin Ireland; ^4^ Department of Pediatrics Trinity College Dublin Ireland; ^5^ Department of Obstetrics and Gynecology Coombe Women's and Infants University Hospital Dublin Ireland; ^6^ Division of Population Health Sciences Royal College of Surgeons in Ireland (RCSI) Dublin Ireland

**Keywords:** clinical teaching programme, congenital heart disease, fetal echocardiography, prenatal diagnosis

## Abstract

**Objectives:**

Improving and maintaining high detection rates for major congenital heart disease (CHD) is a priority for successful prenatal anatomy screening programmes. The primary objective of this study was to evaluate the utility of on‐site multidimensional targeted training in fetal cardiac screening.

**Methods:**

A prospective study evaluating a targeted fetal echocardiography (FE) training programme in two obstetric tertiary care units was established. The programme was designed and approved by a dedicated fetal cardiology team. The one‐day intervention involved supervised hands‐on sonography and a prescribed educational programme for sonographers and doctors. The programme was completed by 23 trainees. Evaluation of the programme involved pre and post‐training questionnaires and retrospective evaluation of cardiac images from 10 randomly‐selected anatomy screening examinations performed by each trainee before and after training. Images were assessed for both technical quality and 6 priority elements of cardiac screening taught in the programme. The results were described with summary statistics and non‐parametric tests.

**Results:**

1850 fetal cardiac images obtained before and after training by 21/23 trainees were assessed. Significant improvement was seen in the priority cardiac views obtained, for example from 24%(5/21) to 86%(18/21) for bifurcating pulmonary artery (*P*‐value 0.001), from 33%(7/21) to 86%(17/21) for differential atrio‐ventricular valve insertion (*P*‐value 0.001), In addition, technical image quality improvements were observed following training as well as sonographer confidence.

**Conclusion:**

A targeted FE training programme can improve understanding of the priority elements required to exclude major CHD in prenatal screening, improve image quality, and sonographer confidence in cardiac screening.

## 1 INTRODUCTION

Prenatal detection of major congenital heart disease (CHD) is one of the primary goals of fetal anatomy screening programmes yet detection rates remain highly variable across the world.^1^, ^2^ Less favorable outcomes are reported in infants diagnosed postnatally, particularly those delivered remote from a tertiary care centre.^3^, ^4^ Studies have demonstrated that the introduction of national screening programmes and targeted training programmes have resulted in greatly improved detection rates.^5^, ^6^, ^7^ Overall, detection rates have not kept pace with increased efforts and improvements in equipment, knowledge and updated cardiac screening guidelines.^8^, ^9^ This suggests that the basic training and standard views alone will not improve screening and that further targeted training and ongoing education may be beneficial. The reported availability and quality of postgraduate training in obstetric and gynecologic ultrasound are also highly variable. A pan‐European survey in 2017 identified dissatisfaction among trainees across 26 countries, with no theoretic or structured practical training available in 16 countries.^10^

In 2021, we conducted a root cause analysis, by way of a national survey across the 19 maternity units in Ireland, to inform the design of this targeted training programme to address the needs of sonographers.^11^ The objective of this study was to design, implement and evaluate the effectiveness of a multi‐dimensional fetal cardiac screening training programme with the aim of improving standards in fetal cardiac screening.

## 2 METHODS

### 2.1 Location and ethical considerations

This study was conducted between September 2022 and March 2024 across two tertiary care maternity sites in Dublin, Ireland (The Rotunda Maternity Hospital and The Coombe Women's and Infants' University Hospital). Both sites combined, deliver 15 500 babies per year (2023), rendering them the busiest obstetric services in Ireland. Ethics Board approval was sought and granted from both sites to run the training programme and retrospectively analyze pre‐ and post‐training fetal cardiac images (RAG REF: RAG‐2023‐006) (REF: study 002–2024). All trainees volunteered for the training programme and gave written consent. All trainee data were anonymized and collated on Excel spreadsheets for analysis. For evaluation of the programmed using image analysis, no patient data were stored for this study. The analysis of images was retrospective, on cases already delivered at the time of retrospective image review. In the event of a concern regarding the potential for missed pathology, a plan was put in place for pediatric cardiology follow‐up.

### 2.2 Study design

A one‐day targeted FE training programme was designed and approved by the prenatal cardiac team (a fetal medicine doctor, a fetal cardiac sonographer and a pediatric cardiologist). The 23 trainees included 7 doctors (specialist registrars or fetal medicine fellows), 11 midwife sonographers and 2 radiographer sonographers (all with a Master's degree level qualification in obstetric ultrasound) and 3 trainee sonographers who were qualified midwives. One trainee per day attended a dedicated fetal cardiology clinic for the purposes of individualized cardiac ultrasound training. A booklet was designed to complement the practical training, which included instruction on machine settings, optimization of cardiac images, diagrams of fetal cardiac anatomy and circulation, labeled ultrasound images on standard cardiac views, priority elements for CHD screening, extended cardiac views and the use of color Doppler. Information on optimal image acquisition for cardiac screening was sought from an applications' specialist on Voluson MDI E8® machines used in both study sites and widely used across Ireland.

The trainees were already competent in the prescribed five views as recommended by national and international guidelines.^9^, ^12^, ^13^ The programme sought to compliment these standard views but also re‐directed the focus of the fetal cardiac screening examination toward emphasis of the key elements of fetal cardiac anatomy and function that must be considered ‘priority’ for excluding major CHD. Recognition of the priority elements listed below using the mnemonic “BADGES” using color Doppler and acquiring an understanding of the relevance of each element for the adequacy of neonatal circulation was taught. Storage of a cine loop/video clip was encouraged to demonstrate these views.

#### 2.2.1 Priority elements, “BADGES” for cardiac screening

**B = B**ifurcating pulmonary artery.

**A = A**pex‐forming left ventricle.

**D = D**ifferential atrio‐ventricular valves.

**G = G**reat artery connection and proportion.

**E = E**qual filling of the ventricles.

**S = S**mooth (forward) flow across the pulmonary/aortic valves (Figure 1).

**FIGURE 1:**
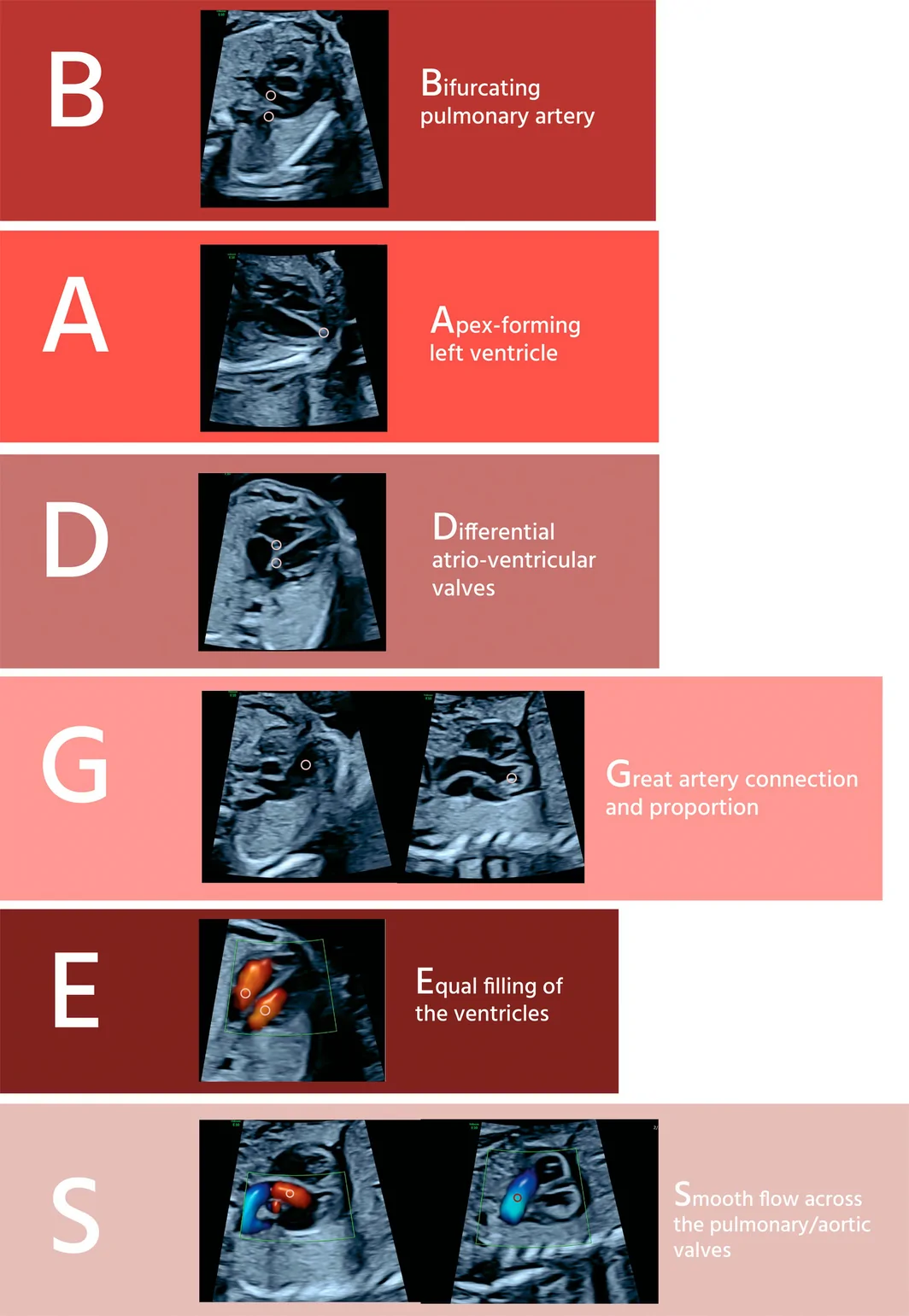
Badges Priority images – Views of the fetal heart in multiple planes using greyscale and color Doppler on transabdominal ultrasound.

The one‐day training course included a prescribed theoretic educational aspect which included the provision of diagrams and videos of fetal cardiac anatomy and fetal circulation, a review of ultrasound images and clips of all critical cardiac pathologies and discussion on how the ‘BADGES’ approach can identify or exclude major CHD (Table 1).

**TABLE 1:** Priority views and major heart disease/pathology identified.

Priority views	Pathology identified
**B** (Bifurcating pulmonary artery committed to right ventricle)	Transposition of the great arteries (TGA)
**A** (Apex‐forming left ventricle)	Hypoplastic left heart disease (HLHD) Hypoplastic right heart disease (HRHD) Shone complex ccTGA Heterotaxy
**D** (Differential atrio‐ventricular valves)	Ebstein anomaly AVSD
**G** (Great artery connection and proportion)	Double outlet right ventricle (DORV) Shone complex Critical aortic stenosis Coarctation of the aorta^a^ (CoA) Critical pulmonary stenosis/atresia Tetralogy of Fallot (TOF) Truncus arteriosus
**E** (Equal filling of the ventricles)	Hypoplastic left heart disease (HLHD) Hypoplastic right heart disease (HRHD) Shone complex Mitral atresia/stenosis Tricuspid atresia/stenosis
**S** (Smooth flow across the pulmonary/aortic valve)	Critical aortic stenosis/atresia Critical pulmonary stenosis/atresia Tetralogy of Fallot (TOF) Coarctation of the aorta (CoA)^a^

Abbreviations: ccTGA, congenitally connected transposition of the great arteries; CoA, coarctation of the aorta; DORV, double outlet right ventricle; HLHD, hypoplastic left heart disease; HRHD, hypoplastic right heart disease; TGA, transposition of the great arteries; TOF, Tetralogy of Fallot.

^a^ CoA – review of the aortic arch also necessary, CoA may not be identified until a later gestation or postnatally.

### 2.3 Evaluation of the training programme

The widely used Kirk‐Patrick model of evaluation^14^, ^15^ where a mixed methods approach of both quantitative and qualitative methods were employed to evaluate the success of the training programme. Quantitative analysis for Kirkpatrick level 2 (proof of learning), level 3 (a change in behavior as a result of training) and level 4 (results to demonstrate outcomes) was completed by image review.

#### 2.3.1 Quantitative – Image analysis/review

The prenatal cardiac team retrospectively reviewed cardiac images up to 3 months before training and at least 3 months after training on the trainees who completed the 1‐day targeted training programme. Images were obtained from the Viewpoint image archiving system on routine anatomy examinations completed by the trainees using a software query to allow for random selection of 10 fetal anatomy cases per trainee to reduce selection bias. Cardiac images reflecting both technical aspects of machine use and the 6 priority (‘BADGES’) elements of the fetal cardiac screen were reviewed and scored by the prenatal cardiac team a fetal medicine expert and fetal cardiac sonographer at both sites, to allow for comparison of pre‐ and post‐training scores. The reviewers were blinded as to which of the 10 anatomy examinations were pre or post training. A score of 1 was assigned if the essential view or technical goal was recorded on any image obtained or 0 if the essential view or technical aspect was not recorded on any images pertaining to an individual anatomy examination. Examination reports were also noted for a normal exam, whether a repeat examination was scheduled or whether referral was made to fetal medicine services for further assessment. Data were collated on Excel and analyzed for 16 questions, 6 BADGES views (Table 2), and 10 technical questions (Table 3).

**TABLE 2:** Successful acquisition and storage of ‘BADGES’ elements, a pre‐ and post‐training analysis of 21 trainees images.

Question:	5 Pre‐training scans per trainee	5 Post‐training scans per trainee	*P* value
B (Bifurcating pulmonary artery)	5/21 (24%)	18/21 (86%)	<0.001
A (Apex‐forming left ventricle)	15/21 (71%)	20/21 (95%)	<0.001
D (Differential atrio‐ventricular valves)	7/21 (33%)	17/21 (81%)	0.004
G (Great artery connection and proportion)	10/21 (48%)	20/21 (95%)	<0.001
E (Equal filling of the ventricles)	17/21 (81%)	20/21 (95%)	0.031
S (Smooth laminar flow across the pulmonary/aortic valve)	7/21 (33%)	17/21 (81%)	<0.001

*Note*: Successful acquisition defined as all elements obtained in at least 4 of 5 examinations. There were between 7 and 10 cardiac images recorded per examination.

**TABLE 3:** Pre‐ and post‐training technical Image analysis on 21 trainees randomly chosen anatomy scans.

Question	5 Pre‐training scans per trainee	5 Post‐training scans per trainee	*P* value
1. Use of cardiac pre‐set	20/21 (95%)	21/21 (100%)	1.000
2. PRF optimized to high, medium or low for maternal BMI	14/21 (67%)	20/21 (95%)	0.010
3. Image magnification >1	21/21 (100%)	21/21 (100%)	0.571
4. Sector width < 25 degrees	6/21 (29%)	16/21 (76%)	<0.001
5.Asingle focal zone at/behind heart	17/21 (81%)	20/21 (95%)	0.028
6.Depth < 11cms	10/21 (48%)	18/21 (86%)	0.008
7. Color box reduced	10/21 (48%)	19/21 (90%)	0.024
8. Color PRF set between 3.5–5.5 kHz	6/21 (29%)	11/21 (52%)	0.020
9. Contrast/tint used	9/21 (43%)	18/21 (86%)	0.002
10. Cine loops/clips obtained	6/21 (29%)	7/21 (33%)	0.110
11. Repeat exam	2/21 (10%)	1/21 (5%)	0.742
12. Referred for fetal medicine review	1/21 (5%)	1/21 (5%)	0.588

*Note*: Successful acquisition defined as all elements obtained in at least 4 of 5 examinations. There were between 7 and 10 cardiac images recorded per examination. BMI, calculated as weight in kilograms divided by the square of height in meters.

Abbreviations: BMI, body mass index; PRF, pulse repetition frequency.

#### 2.3.2 Qualitative – trainee questionnaires

A pre‐training questionnaire was completed by all 23 trainees to assess aspects of prior knowledge and confidence in fetal echocardiography. Following completion of the one‐day training initiative, a tick box sheet was completed to ensure all constituent components of the programme were covered. A post‐training evaluation form was completed by trainees for feedback on all elements of the training programme including the time allocated, quality of the training, topics covered both practically and in the education session. Questions were worded positively and negatively to reduce bias on both questionnaires and a 5‐point Likert scale was used for responses. All questionnaires were completed on the same day as the training. (Table 4).

**TABLE 4:** Pre and post training – Likert questions.

Question	Pre‐training				Post‐training			
	Strongly disagree	Disagree	Agree	Strongly Agree	Strongly disagree	Disagree	Agree	Strongly Agree
I understand fetal circulation/anatomy	0	9 (39%)	8 (35%)	6 (26%)	0	0	7 (32%)	15 (68%)
I feel confident in obtaining an abdominal situs view	0	1 (4%)	11 (48%)	11 (48%)	0	0	1 (4%)	22 (96%)
I feel confident in obtaining a 4‐ chamber view	0	1 (4%)	9 (39%)	13 (57%)	0	0	2 (9%)	21 (91%)
I feel confident in obtaining an LVOT view	0	3 (13%)	7 (30%)	13 (57%)	0	0	3 (13%)	20 (87%)
I feel confident in obtaining an RVOT view	0	2 (9%)	8 (35%)	13 (57%)	0	0	2 (9%)	21 (91%)
I feel confident in obtaining an 3VV	0	4 (17%)	6 (26%)	13 (57%)	0	0	2 (9%)	21 (91%)
I feel confident in obtaining an 3VT view	0	6 (26%)	4 (17%)	13 (57%)	0	0	3 (13%)	20 (87%)
I understand – HLHD/HRHD	1 (4%)	2 (9%)	8 (35%)	12 (52%)	0	0	5 (22%)	18 (78%)
I understand – TOF	2 (9%)	7 (30%)	9 (39%)	5 (22%)	0	0	7 (30%)	16 (70%)
I understand – septal wall defects	0	7 (30%)	11 (48%)	5 (22%)	0	0	6 (26%)	17 (74%)
I understand – TGA	2 (9%)	5 (22%)	9 (39%)	7 (30%)	0	0	7 (30%)	16 (70%)
I understand – DORV	2 (9%)	7 (30%)	10 (43%)	4 (17%)	0	0	8 (35%)	15 (65%)
I understand – PS/AS	2 (9%)	11 (48%)	6 (26%)	4 (17%)	0	0	8 (35%)	15 (65%)
I understand – CoA	2 (9%)	10 (43%)	9 (39%)	2 (9%)	0	0	8 (35%)	15 (65%)
I understand – Arrythmias	2 (9%)	4 (17%)	15 (65%)	2 (9%)	0	0	9 (39%)	14 (61%)
I understand – Ebstein's anomaly	2 (9%)	11 (48%)	7 (30%)	3 (13%)	0	0	11 (48%)	12 (52%)
I understand machine settings	3 (13%)	11 (48%)	9 (39%)	0	1 (4%)	0	10 (43%)	12 (52%)
I feel confident in obtaining an extended heart views	2 (9%)	12 (55%)	5 (23%)	3 (14%)	0	4 (17%)	12 (52%)	7 (30%)
Understand how/when to use Doppler	1 (4%)	6 (26%)	13 (57%)	3 (13%)	0	0	9 (41%)	13 (59%)

Abbreviations: 3VV, three vessel view; 3VT, three vessel and trachea view; AS, aortic stenosis; CoA, coarctation of the aorta; DORV, double outlet right ventricle; HLHD, hypoplastic left heart disease; HRHD, hypoplastic right heart disease; LVOT, left ventricular outflow tract; PS, pulmonary stenosis; RVOT, right ventricular outflow tract; TGA, transposition of the great arteries; TOF, Tetralogy of Fallot.

### 2.4 Statistical analysis

The 10 anatomy examinations for image evaluation per trainee, both pre‐training (5) and post‐training (5), were analyzed using generalized estimating equations and a robust sandwich estimator for variance, as a repeated measures analysis of a binary outcome. *P*‐values for differences between pre‐training and post‐training from these models are presented, for each question. A Likert scale was used to compare pre‐ and post‐training trainee satisfaction and self‐evaluation questionnaire items.

For ease of interpretation of results, “strongly disagree” and “disagree” were combined simply as “disagree”. Similarly, the two agree categories were combined.

## 3 RESULTS

All 23 trainees (17 sonographers and 6 doctors) completed the practical ultrasound training and the pre and post‐training evaluation questionnaires. The theoretic educational aspect of the training was completed by 22/23 trainees and 21/23 had sufficient images available on the Viewpoint® MDI image archiving system for analysis pre‐ and post‐training. The remaining two trainees were junior doctors who had moved to other hospitals shortly after training and did not have sufficient cardiac examinations completed for inclusion in image analysis.

On the 10 randomly chosen (5 pre‐training and 5 post‐training) anatomy examinations on 21/23 trainees, 210 anatomy scans with over 1850 cardiac images, including video clips, were reviewed bythe prenatal cardiac team. Each anatomy scan had between 7 and 10 cardiac images stored. Intra observer variations were not seen between any of the 3 prenatal cardiac team reviewers across both sites. Three out of the 210 randomly chosen anatomy exams had cardiac abnormalities identified and all patients were referred at the time of the scan to fetal medicine services. These were excluded from this analysis as normal BADGES cardiac views could not be appreciated or scored for the purpose of this review. Other examinations were chosen in lieu of these for analysis. No missed cardiac pathology was noted on image analysis.

Image analysis demonstrated a significant increase in the satisfactory acquisition of 6 priority ‘BADGES’ elements in at least 4 of 5 normal anatomy examinations pre and post training for each trainee. (Figure 2). Improvement in the acquisition of each key component of the ‘BADGES’ approach is demonstrated in Table 2. For example, 5/21(24%) trainees demonstrated view B, “a bifurcating pulmonary artery” image in their pre training images, and this improved to 18/21 (86%) on post training image review. (*P*‐value <0,001) Results Tables 2 and 3 are proof of achieving level 4 (results to demonstrate outcomes) in the kirkpatrick model of learning.^14^

**FIGURE 2:**
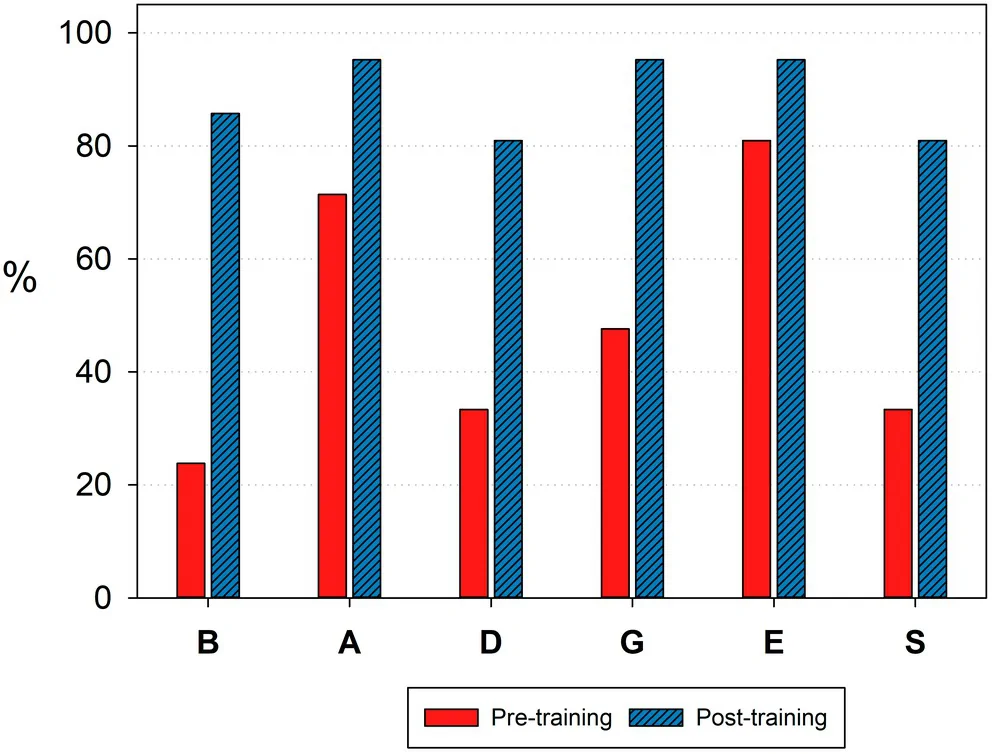
Pre‐ and post‐training inclusion of all 6 ‘BADGES’ elements, for complete prenatal cardiac screen on at least 4 out of 5 exams.

The majority of trainees, 17/21 (81%) had 5 or more years' experience while just 4/21 (19%) had fewer than 3 years' experience so no statistically significant difference was in performance was noticed across the group nor across the sites.

Technical image quality was analyzed on the same trainee images pre and post training Although technical image quality was good in many aspects pre‐training some aspects greatly improved following training such as “decreasing the sector width <25”. Only 6/21 (29%) trainees demonstrated this on anatomy screening exams pre‐training improving to 16/21 (76%) trainees in anatomy examinations post‐training, (Figure 3) which demontrates level 3 (a change of behavior as a result of training) in the kirkpatrick model of learning.^14^

**FIGURE 3:**
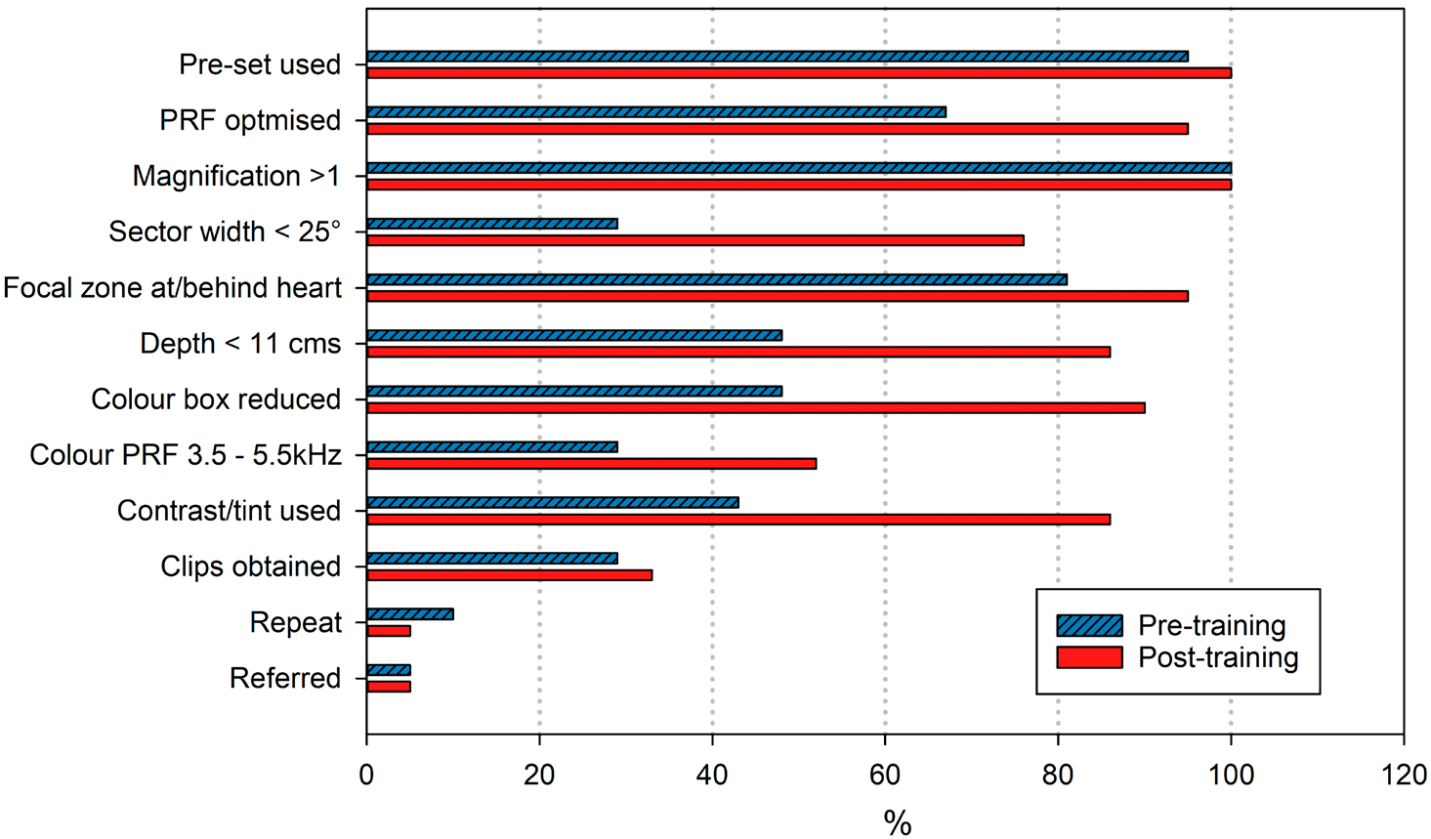
Pre‐ and post‐training technical Image quality on 4 out of 5 exams. PRF, Pulse repetition frequency.

Trainee self‐evaluation questionnaires (Table 4) demonstrated improved trainee confidence and knowledge (Kirkpatrick level 2‐ proof of learning) across all pathologies particularly for cardiac abnormalities other than single ventricle pathology. Confidence in obtaining the 5 standard cardiac views and in recognition of HLHD/HRHD, unsurprisingly was very high in pre‐training questionnaires in this cohort of qualified practitioners. A preference for ongoing training beyond the pre‐specified one‐day intensive training programme was declared by 39% of respondents. 96% (22/23) agreed that the objectives of the training were met in the programme.

## 4 DISCUSSION

The vast majority of CHD cases occur in low‐risk pregnancies, with 90% of affected pregnancies having no risk factors to justify specialist targeted screening echocardiography.^16^ Therefore, development and dissemination of expertise in fetal cardiac screening among fetal sonographers, tasked with identifying cardiac disease in an unselected low‐risk cohort, is of paramount importance in securing high detection rates for major CHD. Studies examining low prenatal detection of CHD cite a lack of targeted training, accessible fetal medicine support, low levels of operator skill, geographical constraints and patient factors such as maternal obesity and suboptimal fetal position as contributing factors.^2^, ^3^, ^4^, ^10^, ^11^, ^17^, ^18^

A study in the Netherlands in 2021 demonstrated that the principal determinant of missed CHD at the mid‐trimester anatomy scan was inadequate quality of fetal cardiac imaging.^18^ Owing to the operator‐dependent nature of prenatal ultrasound, such challenges pose barriers to optimal screening performance. High maternal BMI is also having an impact on maternity care including screening performance.^19^ In addition, resultant repetitive strain in sonographers has become a problematic occupational health issue.^20^ Despite these constraints, sonographers experience increased levels of pressure to deliver excellence in excluding fetal pathology.^11^ Heightened expectations in turn may lead to a greater tendency to recall for repeat examinations and unnecessary referral to specialist services. Although not analyzed in this study, additional training in cardiac screening may also help reduce the requirement for repeat cardiac examinations and/or unnecessary referrals for specialist review. This aspect may be examined in future analyses.

Updated international guidelines that describe a gold standard for prenatal cardiac screening (ISUOG 2023)^9^ include detailed descriptions of fetal cardiac examination including the five standard cardiac views (4 chamber, LVOT, RVOT, 3VV, 3VT) required for satisfactory prenatal clearance of the fetal heart. While the use of color Doppler and cine loop acquisition is encouraged, it is not considered a pre‐requisite for completion of the screening examination, and therefore critical valvular stenosis, dysplasia or regurgitation may be missed. Such valvular abnormalities hold the potential to deteriorate in the early neonatal period. We acknowledge that universal incorporation of color Doppler and capturing the ‘BADGES’ priority elements as described in our study may also not identify all major pathology, including TAPVD (Total anomalous pulmonary venous drainage) and IAA (Interrupted aortic arch). The priority elements described in our training programme are, however, designed to identify the majority of major CHD in a sonographer‐provided non‐specialist screening setting, particularly where the examination is challenging and the classic five prescribed ISUOG views are difficult to obtain. Our targeted training programme described may be useful in complimenting on‐going training needs of qualified sonographers and doctors hoping to improve skills and confidence in examining the fetal heart and improve detection rates for critical CHD.

It has been suggested by Yagel, that detection rates for CHD have not kept pace with improvements in ultrasound equipment and technology.^8^ Solutions suggested for improving CHD standards included ongoing auditing of sonography images, greater support in smaller sites, extra training, in addition to development of artificial intelligence (AI) tools for fetal cardiac screening^8^ Ongoing audit, while essential for determination of quality assurance, will not in its own right lead to improved performance unless accompanied by interventions intended to improve standards. While targeted training programmes can result in improved prenatal detection of CHD, details of such training are not commonly described. Key objectives for any training programme relate to improved staff performance, meeting the needs of patients, and applicability to other centres involved in service delivery.^14^

A strength of our study is that we evaluated the results with both image review and trainee feedback as the success of any training programme can only be measured by reviewing performance before and after training. Our study reports increased confidence levels in machine use, knowledge of cardiac pathology as well as improvement in cardiac image acquisition of our priority elements post training. Repeated testing of trainees in other clinical areas suggests that theoretical knowledge is retained 6 months after a training programme, while newly acquired clinical skills may be lost at a faster rate.^21^, ^22^ This observation supports the theory that ongoing and repeating training programmes are important not only for maintenance of knowledge and skills in that area but also for translation to a behavior that can be measured to improve clinical outcomes.^15^ A review of images, knowledge and skills on the same trainees therefore would be beneficial in the future.

Limitations of this study include, a small number of trainees, 23 doctors and sonographers were trained and assessed for this programme, however a large number of their cardiac images (over 1850) overall were reviewed for data analysis. A strength of the study is that the prenatal cardiac team were unaware which examinations were obtained pre and post training when reviewing cardiac images from the anatomy examinations at both sites.

Our targeted FE training programme has proven the importance not only of improving knowledge, confidence and skills in prenatal cardiac screening but also how that can be translated into a behaviour to improve clinical practice.

We hope to continue to develop this training programme, and offer it to other sonographers and doctors across Ireland in order to improve practical ultrasound skills and knowledge in the prenatal detection of CHD.

## AUTHOR CONTRIBUTIONS

Conception/design of the work: F.C., F.B., O.F., C.L. Data collection: F.C., F.B., Data analysis and interpretation: F.C., F.B., Z.M., P.D., F.M. Drafting the article: F.C. Critical revision of the article: F.B., O.F., Z.M., P.D. and F.M. Final approval of the version to be published: F.C., F.B., O.F., F.M. All authors read and approved the final manuscript.

## FUNDING INFORMATION

The Rotunda Foundation, a charitable organization provided a small educational grant to cover printing costs of the booklet.

## CONFLICT OF INTEREST STATEMENT

The authors declare that they have no conflict of interests.

## ETHICS STATEMENT

All data were collected in accordance with GDPR principles and the Declaration of Helsinki. This study was approved by the Rotunda Advisory Group (RAG REF: RAG‐2023‐006) and by The Coombe Women's and Infants University Hospital ethics board (REF: study 002–2024) prior to commencement of data collection.

## CONSENT FOR PUBLICATION

Individual staff consent was obtained, and data were de‐identified prior to collation and storage.

## Data Availability

Raw Data are stored on a database in the Rotunda Hospital, all data analyzed are included in this published article.
